# Potential Inhibitory Effect of LED-Sourced Red Light Therapy on Ocular Growth in Normal and Myopic Chicks

**DOI:** 10.3390/ijms27125427

**Published:** 2026-06-16

**Authors:** Fengjuan Yu, Kit-Ying Choy, Jingfang Bian, Samantha Sze-Wan Shan, Chi-Ho To, King-Kit Li, Jie Lin, Jingsong Huang, Bo Wang, Dennis Yan-Yin Tse, Rachel Ka-Man Chun, Thomas Chuen Lam

**Affiliations:** 1 Centre for Myopia Research, School of Optometry, The Hong Kong Polytechnic University, Kowloon, Hong Kong; fengjyu@polyu.edu.hk (F.Y.); kitying.choy@polyu.edu.hk (K.-Y.C.); jingfang.jf.bian@polyu.edu.hk (J.B.); samantha.shan@polyu.edu.hk (S.S.-W.S.); chi-ho.to@polyu.edu.hk (C.-H.T.); kk.li@polyu.edu.hk (K.-K.L.); thomas.c.lam@polyu.edu.hk (T.C.L.); 2 Research Centre for SHARP Vision (RCSV), The Hong Kong Polytechnic University, Kowloon, Hong Kong; 3 Centre for Eye and Vision Research (CEVR), Units 901-903, 17W, Hong Kong Science Park, Pak Shek Kok, Shatin, Hong Kong; 4 Oxford Suzhou Centre for Advanced Research (OSCAR), University of Oxford, Suzhou 215123, China; jie.lin@oxford-oscar.cn (J.L.); jingsong.huang@oxford-oscar.cn (J.H.); 5 State Key Laboratory of Ultra-Precision Machining Technology, Department of Industrial and Systems Engineering, The Hong Kong Polytechnic University, Kowloon, Hong Kong; wayne.wang@polyu.edu.hk

**Keywords:** chick, red light, lens-induced myopia, LED, myopia control

## Abstract

Repeated low-level red light (RLRL) has been reported to control myopia progression clinically. Given safety concerns with laser sources, light-emitting diodes (LED)-sourced red light represents a promising alternative. This study investigated the effects of LED-sourced red light (RL) on cellular response in vitro and ocular growth in normal and lens-induced myopic chicks. In vitro, the mouse photoreceptor 661W cell line was exposed to 625 and 664 nm LED-sourced RL (3 min, twice daily) for 3 days, and cytochrome c oxidase (CCO) activity and cell viability were assessed. In vivo, chicks were randomly assigned to normal visual conditions or monocular −5D lens-induced myopia (LIM). Treatment groups received 664 nm LED-sourced RL (30 min, twice daily) at low, moderate, or high intensities for 10 days. In vitro, LED-sourced RL at 664 nm more effectively activated CCO and enhanced cell viability in 661W cells than RL at 625 nm and white light. In vivo, low-intensity RL exposure of 10 days significantly inhibited vitreous chamber depth (VCD) and axial length (AL) elongation compared to the normal light group (*p* < 0.05) in normally growing chicks but showed no significant effect in LIM eyes. By contrast, moderate- and high-intensity RL exposure for 10 days attenuated myopia progression in LIM eyes, as reflected by slower VCD and AL elongation and less myopic shift, compared to the normal light group (all *p* < 0.05). Notably, high-intensity RL also protected the untouched fellow eyes of the LIM chick models against myopic shift and excessive elongation. LED-sourced RL at 664 nm was effective in activating CCO, reducing apoptosis, and promoting cell viability. In chick models, it can also inhibit ocular growth in both normally growing and −5D lens-induced myopic chicks.

## 1. Introduction

Myopia (nearsightedness) is a prevalent refractive error affecting a substantial proportion of the global population. Risk of vision-threatening complications increases steeply with higher intensities of myopia, including retinal detachment, myopic maculopathy, and glaucoma [1,2]. Early-onset myopia in childhood is of particular concern, as it increases the likelihood of progressing to high myopia, underscoring the critical need for effective intervention strategies during developmental years. Current approaches to myopia control primarily include pharmacological agents, optical interventions, and behavioral modifications aimed at visual environment management [3].

Photobiomodulation (PBM), a non-invasive therapy, has recently gained attention for its potential role in regulating ocular growth [4]. Red light (RL) therapy (600–700 nm) is a common PBM modality, whose primary chromophore is mitochondrial cytochrome c oxidase (CCO). Absorption of RL is hypothesized to enhance energy metabolism and trigger signaling cascades involving nitric oxide and reactive oxygen species [4,5,6,7,8]. Notably, clinical studies support the efficacy of laser-sourced repeated low-intensity red-light therapy (RLRL) for controlling myopia progression in children, demonstrating reduced axial elongation [9,10] with a good safety profile over two years [11,12]. The 2025 Myopia Consensus Statement released by the World Society of Paediatric Ophthalmology and Strabismus (WSPOS) noted the effectiveness of RLRL as well as the lower incidence of side effects (0.088 per 100 patient-years) than spectacles for myopia reduction (0.22 per 100 patient-years) and low-dose atropine (7.32 per 100 patient-years). However, the long-term retinal safety of repeated at-home use of laser devices, including potential thermal damage and photochemical risks, remains a concern [13,14,15]. This has prompted the exploration of alternative light sources, including light-emitting diodes (LEDs), which generate negligible heat and may offer a safer profile [16,17].

Emerging evidence suggests that LED-sourced RL may also suppress the development of myopia in young human subjects [18], indicating that the anti-myopic effect is not exclusive to laser sources. Retina tissue isolated from fresh porcine eyes was shown to receive a large proportion of light transmission under both 660 nm red LED and 654 nm red laser irradiation, with more light transmission observed for the LED source [19]. Another study concluded that LED could replace lasers for PBM application without a significant worsening of the results [20]. These studies collectively indicate that wavelength, rather than the level of coherence of light, may be the critical therapeutic factor.

Findings from animal models strongly demonstrated that chromatic RL is pivotal to the development of ocular growth. In contrast, animal studies investigating the effects of monochromatic red light on ocular development yielded inconsistent results, which were largely attributable to inherent species differences in ocular structure, retinal photoreceptor distribution, and neural pathways regulating eye growth. Generally, birds [21], mice [22], and fish [23] tend to develop myopic tendencies under long-wavelength light and hyperopic tendencies under short-wavelength light. However, several mammalian models with more complex visual systems, rhesus monkeys [24], and tree shrews [25,26] showed a promoting effect on hyperopic shifts when subjected to long-wavelength light exposure. Studies on guinea pigs have shown the opposite shift under red light. RL with LED sources at 600 nm and 760 nm with full-day exposure showed promoting myopia effect [27,28], while recent studies with laser-sourced RL at 650 nm (2.23 mW/cm^2^, 3 min for two sessions) and 710 nm (1.67 mW/cm^2^, 15 min × 3 sessions/day) showed suppressing myopia effect in FDM guinea pig models, accompanied with increased choroidal thickness [29,30]. In addition, a significant correlation has been shown between light intensities and the progression of myopia, a pattern consistently observed in children and chick models [31,32]. These findings underscore the critical importance of light sources and parameters in both basic research and potential clinical translation of light-based myopia control strategies.

A primary mechanism underlying red light involves the enhancement of mitochondrial function via the activation of mitochondrial cytochrome c oxidase (CCO) activity. CCO serves as a key mediator in photobiomodulation therapy for myopia and represents a potential target of RL intervention [7,33]. RL within the 600–860 nm range is absorbed by CCO, stimulating ATP synthesis and thereby improving cellular viability and physiological performance [34]. Consistently, RL has been widely reported to significantly increase CCO both in vivo and in vitro [33,35]. Given the critical role of CCO in mitochondrial function and photoreceptor homeostasis, the 661W photoreceptor cell line was chosen for the present study. Since avian photoreceptor cell lines are not commercially available, the mouse-derived 661W cell line has been validated for assessing retinal mitochondrial responses to light stimulation [36]. Therefore, 661W cell lines were employed in this study to characterize red light-evoked cellular responses and compare the effects of two representative RL wavelengths.

To date, no study has systematically explored the dose-dependent effects of LED-sourced monochromatic RL on the process of normal ocular emmetropization and the development of lens-induced myopia (LIM). Given that the chick model is the most widely used, well-established, and highly reliable classic animal model for myopia research [37,38] and has a large spectral range of sensitivity from 370 nm to 680 nm [38], this study adopted this model to systematically investigate the intervention efficacy at different intensity gradients in regulating ocular emmetropization and the development of experimental myopia. We hypothesize that LED-sourced red light may suppress emmetropization and retard myopia progression via a mechanism involving mitochondrial activation. Driven by this hypothesis, this study aims to evaluate the cytoprotective and CCO-activating effects of red light in 661W cells and determine its efficacy in modulating both ocular emmetropization and lens-induced myopia development in a classic chick model. It is expected to provide experimental evidence for exploring the mechanism of light-mediated myopia control and optimizing intervention parameters.

## 2. Results

### 2.1. Exposure to 664 nm Red Light Promoted Cell Viability Through Activation of CCO and Inhibition of Apoptosis

**Figure 1:**
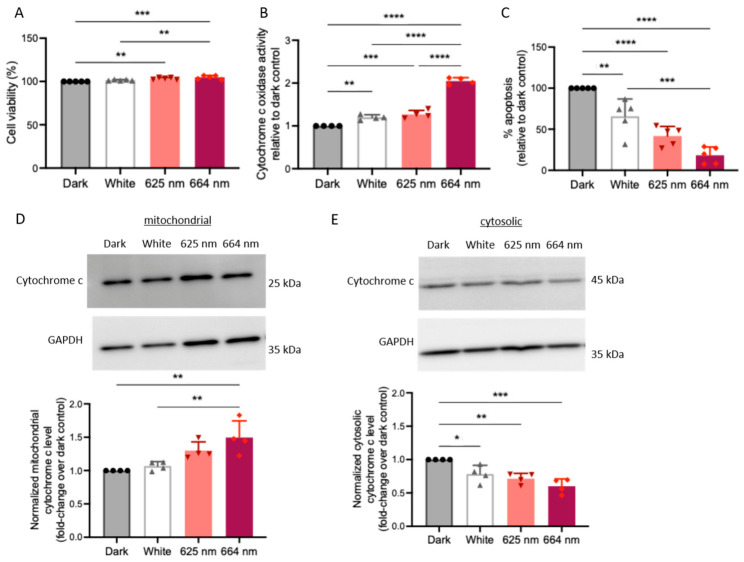
The assessment of (**A**) cell viability (*n* = 5), (**B**) CCO activity (*n* = 4), (**C**) apoptosis (*n* = 4), (**D**) a representative of four individual Western blots of cytochrome c expression in mitochondrial fractions and the normalized cytochrome c protein level (*n* = 4), and (**E**) a representative of four individual Western blots of cytochrome c expression in cytosolic fractions and the normalized cytochrome c protein level (*n* = 4) after light exposure in 661W cells. Data represent mean ± SD. The statistical significance was assessed by one-way ANOVA with Bonferroni post hoc test (* *p* < 0.05, ** *p* < 0.01, *** *p* < 0.001, **** *p* < 0.0001).

Three-day exposure to 625 and 664 nm RL significantly promoted cell viability by 3.3 ± 1.2% (*p* < 0.01) and 4.8 ± 2.1% (*p* < 0.001; Figure 1A), respectively, and had no adverse effects on cell morphology. Normal white light did not affect cell viability, confirming the observed outcome was caused by the exposure to RL of specific wavelengths (625 and 664 nm), but not other factors such as the heat generated from the light source. Red light exposure increased CCO activity in all RL-exposed cells when compared to the dark control. RL at 664 nm was more effective in activating enzyme activity, which increased it by 2.0-fold (*p* < 0.001), whereas 625 nm and white light increased it by 1.2-fold (*p* < 0.001) and 1.3-fold (*p* < 0.01), respectively, when compared to the dark control (Figure 1B). RL at 664 nm exhibited a superior effect in reducing apoptosis, achieving a reduction of 82% (*p* < 0.0001; Figure 1C). RL at 625 nm also significantly reduced apoptosis by 58% (*p* < 0.0001), while white light significantly reduced it by 34% (*p* < 0.01).

Exposure to 664 nm LED-sourced RL led to significantly increased retention of cytochrome c in mitochondria (1.5-fold; *p* < 0.01; Figure 1D) and reduced its release to the cytosol (0.6-fold; *p* < 0.001; Figure 1E), when compared to the dark control. RL at 625 nm had a similar, but less pronounced, effect than 664 nm. RL exposure also protected all light-exposed cells from apoptosis when compared to the dark control. Overall, the findings demonstrated that RL at 664 nm demonstrated a more potent effect than 625 nm in activating CCO activity and preventing the release of cytochrome c from mitochondria to cytosol, which subsequently reduced apoptosis and promoted cell viability.

### 2.2. Low-Intensity Red Light Inhibits Ocular Elongation in Normal Chicks

**Figure 2:**
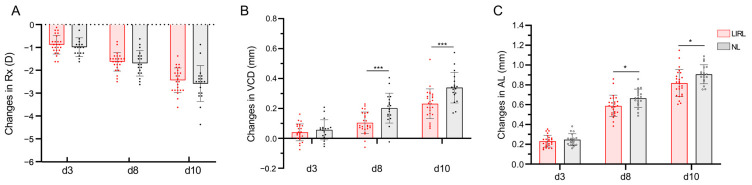
Changes in (**A**) Rx, (**B**) VCD, and (**C**) AL in normal chicks following LIRL treatment. Data of individual cases in each group are shown in dots. Differences in LIRL-treated eyes and NL groups were compared using two-way repeated ANOVA (* *p* < 0.05, *** *p* < 0.001).

In Exp. 1, no significant differences were observed in body weights (BW), refractive error (Rx), and all ocular biometric parameters between groups at baseline. After 10 days, low-intensity red light (LIRL) exposure did not significantly affect changes in BW or Rx compared to the normal light (NL) group (Figure 2A). However, the increase in vitreous chamber depth (VCD) was significantly smaller in the LIRL group after 8 days (0.10 ± 0.07 mm vs. 0.20 ± 0.10 mm) and 10 days (0.23 ± 0.10 mm vs. 0.34 ± 0.10 mm; *p* < 0.05, Figure 2B) compared to NL group, representing inhibitory percentages of 29.6% (d3), 48.4% (d8), and 31.8% (d10), respectively. Axial length (AL) elongation matched well with VCD changes (Figure 2C), with less inhibitory percentages of 6.1% (d3), 11.5% (d8), and 9.9% (d10), respectively.

### 2.3. Low-Intensity Red Light Is Ineffective Against Lens-Induced Myopia in Chicks

**Figure 3:**
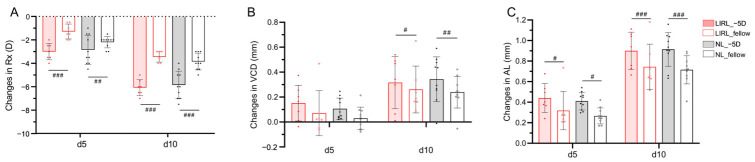
Changes in (**A**) Rx, (**B**) VCD, and (**C**) AL following LIRL treatment. Data of individual cases in each group are shown in dots. Differences in lens-induced eyes and fellow eyes were compared using a paired *t*-test (# *p* < 0.05, ## *p* < 0.01, ### *p* < 0.001). No differences in LIRL-treated eyes and NL groups were found using two-way repeated ANOVA.

In Exp 2, myopia was successfully induced by −5D lens wear with significant myopic shifts, increased VCD and AL in lens-induced eyes compared to fellow eyes (*p* < 0.05; Figure 3A–C). However, LIRL treatment did not induce significant differences in Rx, VCD, or AL changes compared to the NL group at any time (*p* > 0.05).

### 2.4. Moderate- and High-Intensity Red Light Suppressed Myopia Progression in Lens-Induced Myopic Chicks

**Figure 4:**
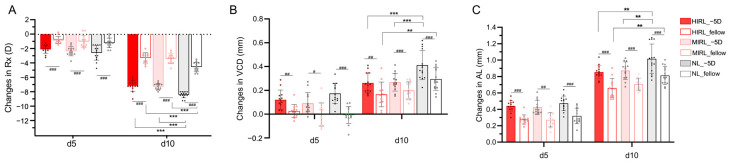
Changes in (**A**) Rx, (**B**) VCD, and (**C**) AL following HIRL and MIRL treatment. Data of individual cases in each group are shown in dots. Differences in lens-induced eyes and fellow eyes were compared using a paired *t*-test (# *p* < 0.05, ## *p* < 0.01, ### *p* < 0.001). Differences in HIRL- and MIRL-treated eyes and NL groups were compared using two-way repeated ANOVA ** *p* < 0.01, *** *p* < 0.001).

In Exp 3, myopia was significantly induced by −5D lenses in all groups (*p* < 0.05; Figure 4A). After 10 days, both high-intensity red light (HIRL) and moderate-intensity red light (MIRL) treatments significantly reduced the myopic shift in LIM eyes (HIRL: −7.17 ± 0.76D; MIRL: −7.27 ± 0.44D) compared to the NL group (−8.43 ± 0.42D; *p* < 0.05). Protective effect was also observed in the untreated fellow eyes of the HIRL and MIRL groups (HIRL: −3.29 ± 0.71D; MIRL: −3.42 ± 0.68D) compared to NL fellow eyes (−4.55 ± 0.65D; *p* < 0.05; Figure 4A). The corresponding inhibitory percentages in LIM eyes were 14.9% (HIRL) and 13.7% (MIRL), while percentages in fellow eyes were 27.8% (HIRL) and 24.8% (MIRL), compared to the NL groups. No significant differences were observed between HIRL and MIRL groups.

At day 10, VCD and AL elongation in LIM eyes were significantly reduced in both HIRL (VCD: 0.26 ± 0.08 mm; AL: 0.85 ± 0.08 mm) and MIRL (VCD: 0.27 ± 0.07 mm; AL: 0.87 ± 0.11 mm) groups compared to the NL group (VCD: 0.41 ± 0.12 mm; AL: 1.02 ± 0.18 mm; and *p* < 0.05, Figure 4B,C). In fellow eyes, significant inhibition of VCD and AL elongation was observed only in the HIRL group (VCD: 0.17 ± 0.10 mm; AL: 0.66 ± 0.11 mm) compared to the NL group (VCD: 0.29 ± 0.10 mm; AL: 0.81 ± 0.11 mm; and *p* < 0.05; Figure 4B,C). The corresponding inhibitory percentages in VCD and AL in LIM eyes were 36.2% and 15.9% (HIRL), 34.8% and 14.2% (MIRL), and 43.8% and 18.8% (HIRL), and 32.5% and 13.1% (MIRL) in fellow eyes, compared to the NL group.

### 2.5. Effects of Red Light Treatment on Choroidal Thickness and the Anterior Segment

**Figure 5:**
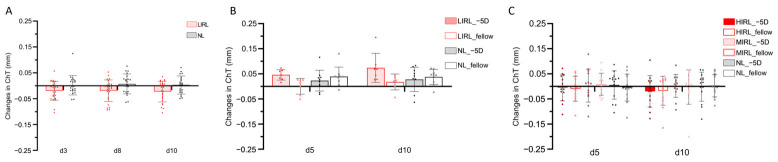
Changes in ChT following different RL treatments. (**A**) LIRL treatment to normal chicks. (**B**) LIRL treatment to LIM chicks. (**C**) HIRL and MIRL to LIM chicks for 10 days. Data of individual cases in each group are shown in dots. Data represent mean ± SD. Differences in RL-treated eyes and NL groups were compared using two-way repeated ANOVA (mean ± SD).

At baseline, choroidal thickness (ChT) did not differ significantly across the three experiments (*p* > 0.05). LIRL, MIRL, or HIRL treatment did not induce significant ChT changes relative to respective NL groups in any of the experiments (*p* > 0.05; Figure 5). In addition, no notable ChT differences were detected in Exp. 1–3 for either LIM or fellow eyes across all intervention groups (*p* > 0.05).

For the anterior segment, only LIRL induced increased elongation in lens thickness at day 8 (*p* < 0.05). However, this effect was no longer statistically significant by day 10 in normal chicks. None of the interventions (LIRL, MIRL, and HIRL) exerted a notable impact on anterior segment parameters, including anterior chamber depth and lens thickness in LIM models.

## 3. Discussion

This in vitro study demonstrated that LED-sourced 664 nm showed an effective and potent effect in activating CCO activity and reducing apoptosis. Cytochrome c oxidase, or Complex IV, is the last protein complex of the mitochondrial electron transport chain, and its activity is essential to energy production to support cell proliferation and activities [39]. It exhibits distinct absorption peaks in the red and near-infrared (NIR) regions, at approximately 650–680 nm in its oxidized state and 710–790 nm in its reduced state [40]. Aligning with the absorption peak of oxidized CCO, 664 nm is more effective than 625 nm in mediating PBM [41]. Although 661W cells exhibit high sensitivity to blue light, they remain responsive to red light [42,43,44] despite the relatively low photon capture efficiency of cone opsins at longer wavelengths. Red to NIR may react with various target receptors, such as copper centers of CCO [45] or nanoscopic interfacial water layers [46]. In either case, CCO is activated to drive the mitochondrial electron transport chain for ATP production and the release of nitric oxide and reactive oxygen species for PBM therapeutic effects. In addition, the effects of 664 nm RL were also tested in human retinal pigmented epithelial ARPE-19 cells (Supplementary Figure S1) and monkey endothelial RF/6A (Supplementary Figure S2), resulting in a consistent activity profile. It indicated that the effects of PBM were not cell-line-specific and might affect a broad range of cells via a universal mechanism. Nevertheless, species differences between mouse and chicken photoreceptors must be considered when interpreting the results [47,48,49].

While not as effective as RL at 664 nm, white light exposure also significantly protected 661W cells from apoptosis and was associated with increased CCO activity and reduced cytosolic cytochrome c levels, suggesting the preservation of mitochondrial integrity. Although not aligned with previous reports which suggested prolonged white light exposure (2500 lux up to 24 h) induced ROS production and apoptosis in 661W cells [50,51], this anti-apoptotic effect of brief, repeated white light exposure is biologically plausible and may reflect a preconditioning phenomenon, in which moderate bright light induces a protective program that renders retinal cells more resistant to subsequent light-induced damages [52,53]. However, further investigations are required to confirm the contribution of preconditioning mechanisms.

As for in vivo studies using chick models, previous studies using monochromatic light exposure mainly reported myopia shift when exposed to RL. One-day-old chicks raised in LED-sourced RL (peak at 641 nm and luminance of 33.37 cd/m^2^) with a 12L/12D cycle caused progressive myopia after 21 days of treatment, while blue light induced chicks to progressive hyperopia. [54] Furthermore, the induced hyperopia by blue light could be reversed to myopia by subsequent red light treatment for another 21 days [54]. In contrast, chicks under RL (665 nm, 48 mW/m^2^) for 12 days with a 12/12 h light/dark cycle exposure remained slightly hyperopic, with no difference from control groups. Additionally, the results of exposure to RL on chicks wearing −4D lenses were not different from those obtained in white light chicks [38]. LIM chick models with 14 h of light exposure, an increase in AL (60 µm) and a decrease in ChT (−51 µm) compared to fellow eye were observed in white light group, while there were no changes in AL (8 µm) and decrease in ChT (−62 µm) in red light (620 nm), with a low illuminance around 0.67 chick lux [21]. Although not significant, chicks without any lenses showed a pattern of shorter elongation of VCD and AL under LED-sourced narrow-band RL (634 ± 15 nm, 250 lux), compared to white light treatment [55]. The latter studies all showed a controlling effect on AL of red light, at least no promoting myopia effect.

Comparison of the present findings with previous chick model studies suggests that variations in light parameters and experimental design may mediate the biological effects of RL. This study employed an optimized experimental configuration to better mimic the power used in clinical studies and refined the setup to deliver stable power to the chick eyes. The current study employed short-duration (30 min), intermittent, direct ocular exposure to narrow-band LED-sourced red light at 664 nm, whereas other studies mainly used long-duration (e.g., 12 h) continuous exposure. Constant RL was previously shown to alter ocular growth rhythms in chicks and induce different retinal signaling pathways [56], which may cause circadian interruption in animals. The posture of chicks lying horizontally beneath the RL source ensures that a more consistent and similar amount of RL enters the eyes of all treated chicks. In contrast, chicks that walked freely in cages with LED lights positioned above the cage received considerably less RL exposure to the eyes, as chicks typically direct their gaze toward the ground. Additionally, the RL of high and moderate intensity in the current study was used. Consistent with this, the documented dose-dependent effects of illuminance and power on treatment outcomes support the critical role of the dosing regimen [31,32,57]. It is worth noting that the ocular parameters of VCD and AL varied between normally raised chicks (Figure 2) and fellow eyes of LIM chicks (Figure 3 and Figure 4). It was primarily attributed to the inherent batch-to-batch variability commonly observed in avian models. To eliminate this confounding factor, all experimental and control groups within each experiment were strictly age- and batch-matched.

Another key finding is the differential efficacy of RL intensity across distinct refractive status groups. LIRL effectively inhibited ocular growth in chicks not subjected to −5D lenses but failed to exert a comparable inhibitory effect in chicks with −5D lenses. A similar pattern has been reported in human studies, where incoherent narrow-band RL (620 ± 10 nm) administered for 10 min was found to be more effective in non-myopic eyes than in myopic eyes. An axial shortening of 71% and 41% was observed in non-myopic and myopic eyes, respectively [18]. Furthermore, moderate- and high-intensity RL demonstrated significant efficacy in inhibiting ocular growth in chicks with −5D lens induction. These observations led to the proposal that the myopic eye may require sufficiently intense RL stimuli to trigger growth-inhibiting signaling pathways. Additionally, the comparable efficacy between MIRL and HIRL suggests a potential ceiling effect of RL intensity on ocular growth inhibition in the chick LIM model. On the other hand, the inhibitory effect on the non-induced fellow eyes of LIM models was exclusively observed under HIRL conditions, whereas LIRL and MIRL elicited no detectable impact. This discovery implies that although both the fellow eyes of the LIM model and the eyes of normally raised chicks were not treated by negative lenses, they possess disparate susceptibilities to red light. Such a divergence may be driven by systemic signaling emitted from the LIM eyes, which subsequently reaches and influences the contralateral eye.

Notably, RL intervention did not trigger significant changes in choroidal thickness in both normal and LIM chicks, whereas increased ChT is a well-documented key correlate of myopia control efficacy in clinical studies [58,59]. A dose-dependent effect on sub-foveal choroidal thickness has been demonstrated in children [31]. Previous studies have yielded inconsistent ChT responses to RL in chick models. A thinner ChT (−62 ± 14 μm) with RL treatment (620 nm; 10 nm bandwidth) compared to white light (−51 ± 10 μm, *p* < 0.001) in chicks was reported [21], while another found no ChT changes in either treated or fellow eyes of dual-power optical lens chick models after 13 days of LED-sourced RL exposure (634 ± 15 nm, 250 lux) [55]. Apart from variations in light source, light intensity, and wavelength, such discrepancies may be attributed to variations in intervention duration. LED-sourced RL treatment (628 ± 10 nm, 424 lux) for 10 days showed no effects, but treatment of 17 days induced significant thickening of ChT in chicks [56]. Clinical data further revealed a time-dependent dynamic change in ChT, which peaked at 1 month (14.755 μm), gradually declined at 3 months (5.286 μm) and 6 months (1.543 μm), and eventually reached 9.089 μm at 12 months [59]. Intervention duration may serve as a key confounding factor, and the dynamic characteristics of ChT responses to RL remain to be further clarified. Moreover, substantial inter-individual variability in ChT was observed in the present study (90% CI: −99 to 81 μm), which may mask subtle changes in ChT. Our pilot experiments on chick models demonstrated that a 30 min exposure to red light induced a transient increase in choroidal thickness. However, this effect vanished after long-term exposure, suggesting that the choroidal response to red light in chicks is primarily an acute, short-term phenomenon. Collectively, the underlying mechanisms driving myopia control in our model may differ from the choroid-mediated pathways typically proposed in clinical RLRL studies. Additionally, the mechanistic link between in vitro mitochondrial responses and in vivo ocular growth inhibition remains inferential rather than directly proven. The mechanism of mitochondrial function at the retinal level in chick models needs to be studied to definitively link these two experimental components in future studies.

One concern of the present study is the potential impact of RL on the pineal gland, which is known to regulate melatonin secretion and participates in circadian rhythm modulation and ocular growth [60]. Actually, RL has weak effects on pineal melatonin secretion compared to short-wavelength light and rarely causes significant suppression compared to white light, especially during daytime exposure [61]. Our short intermittent daytime RL exposure barely disturbed pineal function, which is consistent with previous findings that pinealectomy did not have a significant effect in chick models under a 12L/12D cycle [62]. Thus, its minimal response to our short RL intervention under 12L/12D indicates negligible influence on the present study’s results.

## 4. Materials and Methods

### 4.1. Cell Culture and Illuminance Conditions

The mouse photoreceptor 661W cells were provided by Dr. Muyyad R. Al-Ubaidi [63] and maintained in Dulbecco’s Modified Eagle Medium (DMEM; Gibco, Thermo Fisher Scientific, Waltham, MA, USA) supplemented with 20 µg hydrocortisone 21-hemisuccinate, 40 µg progesterone, 0.032 g putrescine, 40 µL beta-mercaptoethanol, 10% fetal bovine serum (FBS; Gibco), and 1% penicillin-streptomycin (PS; Gibco). Cells were incubated at 37 °C in a humidified atmosphere of 5% carbon dioxide until 70% confluent.

Within the same incubator, cells were incubated with LED-sourced red light at wavelengths of 625 ± 25 nm or 664 ± 34 nm (Ingbright Lighting Co., Ltd., Jiangmen City, China), with the light sources placed 0.5 cm directly under culture plates to ensure consistent light intensity for all cultures. The entire illumination setup was operated within a standard cell culture incubator maintained at 37 °C. Wavelengths of 625 and 664 nm were selected as they closely align with the absorption peaks of cytochrome c oxidase [40]. Two control plates were also incubated at the same time, one placed above a normal white light source (Ingbright Ltd.) to minimize the effects of light exposure and heat produced from the light source on cell responses (white control), and the other fully covered with aluminum foil to prevent light from entering (dark control). Custom-designed black acrylic lids containing multiple small ventilation holes were placed over the entire plate to minimize light leakage across different wavelengths while ensuring sufficient air exchange during incubation. RL irradiance was quantified using a cal-Light 400 calibrated precision light meter (Cooke Corporation, Princeton, NJ, USA) and a PD100-350-TP100 power meter (Changchun New Industries Optoelectronics Technology Co., Ltd., Changchun, China). The illuminance at the plates was 2500 lux, which reports photometric units weighted to the human photopic spectral sensitivity. The corresponding powers at the cell layer were 1.06 mW at 625 nm and 2.50 mW at 664 nm. Cells were exposed to 625 nm RL, 664 nm RL, or white light for 3 min, twice daily (with a 4 h interval) for 3 days, mirroring the clinical RLRL treatment regimen used in children [9]. After light exposure, cells were immediately harvested for subsequent activity assays. The mitochondrial and cytosolic fractions were isolated using a Mitochondrial/Cytosol Fractionation Kit (ab65320; Abcam, Cambridge, UK) according to the manufacturer’s instructions and quantified using the Bio-Rad Protein Assay (5000001; Bio-Rad Laboratories, Hercules, CA, USA).

### 4.2. Measurement of Cell Viability, CCO Activity, Apoptosis, and Western Blot Analysis

Cell viability was determined by the trypan blue dye exclusion test as previously described [64]. The percentage of viable cells was normalized to the dark control. CCO activity was measured using a Cytochrome C Oxidase Assay kit (ab239711; Abcam), and the enzyme activity was normalized to the dark control. Apoptosis was determined using a TUNEL Assay Kit (ab66108; Abcam). Both assays were performed according to the manufacturer’s instructions. The fluorescent signals were quantified by CytoFLEX S flow cytometry (Beckman Coulter, Brea, CA, USA), and the percentage of apoptotic cells was normalized to the dark control.

The mitochondrial (30 µg) and cytosolic fractions (100 µg) in SDS loading buffer (Bio-Rad Laboratories) were loaded onto a 10% SDS-PAGE gel and transferred to a polyvinylidene fluoride (PVDF) membrane (Bio-Rad Laboratories) after separation. The PVDF membrane was incubated with anti-cytochrome c antibody (1:1000) at 4 °C overnight, followed by incubation with goat secondary antibodies conjugated with horseradish peroxidase (1:1000, Thermo Fisher Scientific). Anti-GAPDH antibody (1:1000, Calbiochem, Merck, Burlington, MA, USA) was also included as a loading control for normalization. Protein bands were visualized using the Pierce SuperSignal West Pico Chemiluminescent substrate (Thermo Fisher Scientific), imaged with the ChemiDoc imaging system (Bio-Rad Laboratories), and quantified using ImageJ software version 1.54r.

### 4.3. Animal Models and Experimental Grouping

White leghorn chicks (*Gallus gallus*) were hatched from the SPF eggs (Jinan Poultry Co., Ltd., Jinan, China) and housed in iron cages with dimensions of 60 (L) × 40 (W) × 50 (H) cm under a 12 h light/12 h (12L/12D) dark cycle (lights on from 7:00 AM to 7:00 PM). The housing environment was maintained under controlled temperature (20–22 °C). Food and water were provided ad libitum. All procedures adhered to the ARVO statement for the Use of Animals in Ophthalmic and Vision Research and were approved by the Animal Subjects Ethics Sub-Committee (ASESCs) of the Hong Kong Polytechnic University (No: 21-22/212-SO-R-OTHERS).

At postnatal day 5 (PN5), chicks were randomly allocated into three experimental groups. In Exp. 1, chicks without lenses were divided into an LIRL group (*n* = 25) and a normal light (NL) group (*n* = 18). In Exp. 2, LIM chicks received LIRL (*n* = 7) or NL treatment (*n* = 10). In Exp. 3, LIM chicks were assigned to HIRL (HIRL, *n* = 13), MIRL (MIRL, *n* = 13), or NL (*n* = 14) treatment. For LIM model establishment, a customized negative −5D single-vision polymethyl methacrylate (PMMA) lens was applied monocularly to one eye of all chicks. A hollow annular Velcro strip was used to secure the lens to the feathers surrounding the orbital rim, ensuring the lens center remained aligned with the pupil.

### 4.4. Illuminance Conditions on Chick Models

The LED-sourced RL in chick models exhibited a central wavelength of 664 ± 34 nm and a full width at half maximum (FWHM) of 24 nm. The entering eye power was calculated based on the average chick pupil diameter (2 mm) and the detector diameter of the power meter (9 mm). The irradiance was calculated based on the measured power of the power meter divided by the detector area. Furthermore, the entering eye power of 0.29 mW in the HIRL group was selected based on its frequent use in relevant clinical research [9,10], with calculations performed following the method described by Xiong et al. [65]. The measured illuminance and calculated entering eye power for three RL treatments were as follows: LIRL: 450 ± 22 lux, 0.04 mW; MIRL: 2500 ± 52 lux, 0.15 mW; and HIRL: 6000 ± 85 lux, 0.29 mW. The illuminance of normal light (NL) was maintained at 265 ± 22 lux.

Using chick models, our prior pilot data confirmed that 30 min RL exposure elicited a more pronounced effect than both 15 min and 1 h exposure sessions. Accordingly, RL treatment was administered twice daily for 10 consecutive days, with each session lasting 30 min and at least a 4 h interval. The −5D lenses in LIM eyes were kept on during all RL exposure sessions to avoid recovery effects and all treatments were conducted in darkness to eliminate confounding effects from ambient normal visual input. During each RL treatment session, chicks were gently restrained in a soft tissue wrap and placed horizontally beneath the LED RL source to ensure uniform and consistent light exposure across all subjects and both eyes. Chicks within the same group were treated simultaneously at a fixed distance of 15 cm from the light source, with treatment administered at the same fixed times daily (10:00 AM and 3:00 PM).

Throughout the entire experimental period, the lenses were cleaned and inspected daily to verify proper positioning and integrity. Chicks with dislodged lenses were excluded from the final analysis to avoid biased outcomes due to recovery. To further optimize experimental conditions and minimize non-specific interference, a ventilation fan was installed above the cage to improve air circulation, and the cage temperature inside the cage was monitored and maintained below 25 °C.

### 4.5. Parameters Measurement and Statistical Analysis

Refractive error (Rx) was measured by a streak retinoscope (Heine Beta 200, Heine OptoTecnik, Gilching, Germany), and spherical equivalent was calculated as the sum of spherical power and half of the cylindrical power. Ocular biometric parameters, including VCD, ChT, and AL, were acquired using high-frequency A-scan ultrasonography. The details on the peak labeling and definition of ocular biometrics were described in our previous study [55]. All measurements were performed at low illuminance (<50 lux).

**Figure 6:**
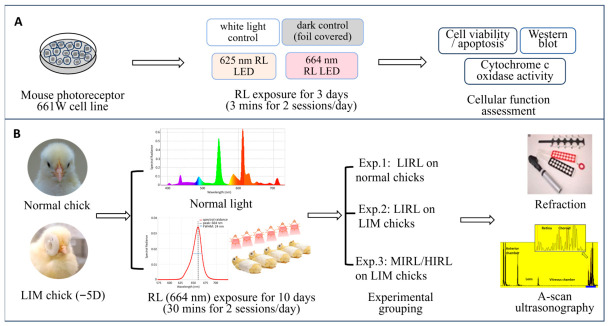
Schematic illustration of (**A**) in vitro study using the 661W cell line and (**B**) in vivo experiments for evaluating the effects of LED-sourced RL. LIRL: low-intensity red light; MIRL: moderate-intensity red light; HIRL: high-intensity red light.

For in vitro data, one-way analysis of variance (ANOVA) with Bonferroni post hoc test was used to compare means among light exposed groups and the dark control. To avoid the impact of growth differences among chicks from different batches on the experimental results, the treated group was compared with the control group from the same batch in Exp. 1 to Exp. 3. A priori power analysis indicated that a minimum sample size of 6 chicks per group provided 80% power to detect a 0.1 mm difference in axial length (SD = 0.1 mm, Cohen’s d = 2.0) at alpha = 0.05. For chicks without lenses, data from both eyes were averaged for subsequent analysis. For chicks fitted with −5D lens, the lens-induced eyes and their fellow eyes were compared to assess the lens induction effect using paired *t*-tests. Changes from baseline in Rx and ocular biometrics were analyzed using two-way repeated-measures ANOVA. To control the risk of multiple comparison inflations arising from multiple groups, longitudinal repeated measurements, bilateral analyses, and various biometric endpoints, conservative Bonferroni post hoc adjustments were strictly applied. Data are represented as mean ± SD, with *p* < 0.05 considered statistically significant. The schematic illustration of the experimental design workflow is summarized in Figure 6.

## 5. Conclusions

In conclusion, LED-sourced RL at 664 nm was demonstrated to exhibit a superior effect and be more effective than RL at 625 nm in activating CCO activity and reducing apoptosis. It represents a potential and non-invasive approach for modulating ocular growth in normally growing and lens-induced myopic chick models. Furthermore, the data suggest a potential ceiling effect, whereby increasing the intensity beyond a certain threshold does not yield additional benefit. Its efficacy is wavelength-specific, intensity-dependent, and varies with refractive status, underscoring the need for precise dosing protocols. While LED sources offer theoretical safety advantages, evaluating their prospective toxicity on retinal tissues remains imperative. Consequently, further studies are warranted to rigorously evaluate the long-term safety of LED-sourced RL and to elucidate its molecular mechanisms in modulating eye growth using chick models. The absence of choroidal thickening in chicks highlights species-specific responses. The differential protective effect on myopic eyes emphasizes the need for precise dosing protocols tailored to the severity and progression of myopia.

## Data Availability

The original contributions presented in this study are included in the article/Supplementary Materials. Further inquiries can be directed to the corresponding authors.

## Supplementary Materials

The following supporting information can be downloaded at https://www.mdpi.com/article/10.3390/ijms27125427/s1.

## Abbreviations

The following abbreviations are used in this manuscript:

AL	Axial length
ANOVA	Analysis of variance
ARPE19	Human retinal pigmented epithelial cells
ARVO	Association for Research in Vision and Ophthalmology
ASESCs	Animal Subjects Ethics SubCommittee
ATP	Adenosine triphosphate
Ascan	Ascan ultrasonography
CCO	Cytochrome c oxidase
CEVR	Centre- for Eye and Vision Research
ChT	Choroidal thickness
DMEM	Dulbecco’s Modified Eagle Medium
FBS	Fetal bovine serum
FDM	Form-deprivation myopia
FWHM	Full width at half maximum
HIRL	High-intensity red light
IOVS	Investigative Ophthalmology and Visual Science
LED	Light-emitting diode
LIM	Lens-induced myopia
LIRL	Low-intensity red light
MIRL	Moderate-intensity red light
OCT	Optical coherence tomography
OSCAR	Oxford Suzhou Centre for Advanced Research
PBM	Photobiomodulation
PMMA	Polymethyl methacrylate
PN5	Postnatal day 5
PS	Penicillin-streptomycin
RL	Red light
RLRL	Repeated low-level red light
RCSV	Research Centre for SHARP Vision
RF6A	Monkey endothelial cells
ROS	Reactive oxygen species
Rx	Refractive error
SD	Standard deviation
SPF	Specific pathogen-free
TUNEL	Terminal deoxynucleotidyl transferase dUTP nick end labeling
VCD	Vitreous chamber depth
WSPOS	World Society of Paediatric Ophthalmology and Strabismus
PVDF	Polyvinylidene fluoride
