# Supplementary material for: Potential Inhibitory Effect of LED-Sourced Red Light Therapy on Ocular Growth in Normal and Myopic Chicks
Source: Int J Mol Sci. 2026 Jun 16;27(12):5427. doi: 10.3390/ijms27125427 (PMC13300664; doi:10.3390/ijms27125427)
Supplement: Supplementary file 1 [file ijms-27-05427-s001.zip › ijms-4327222-supplementary.pdf]

International Journal of
Molecular Sciences

MDPI

Supplementary Material

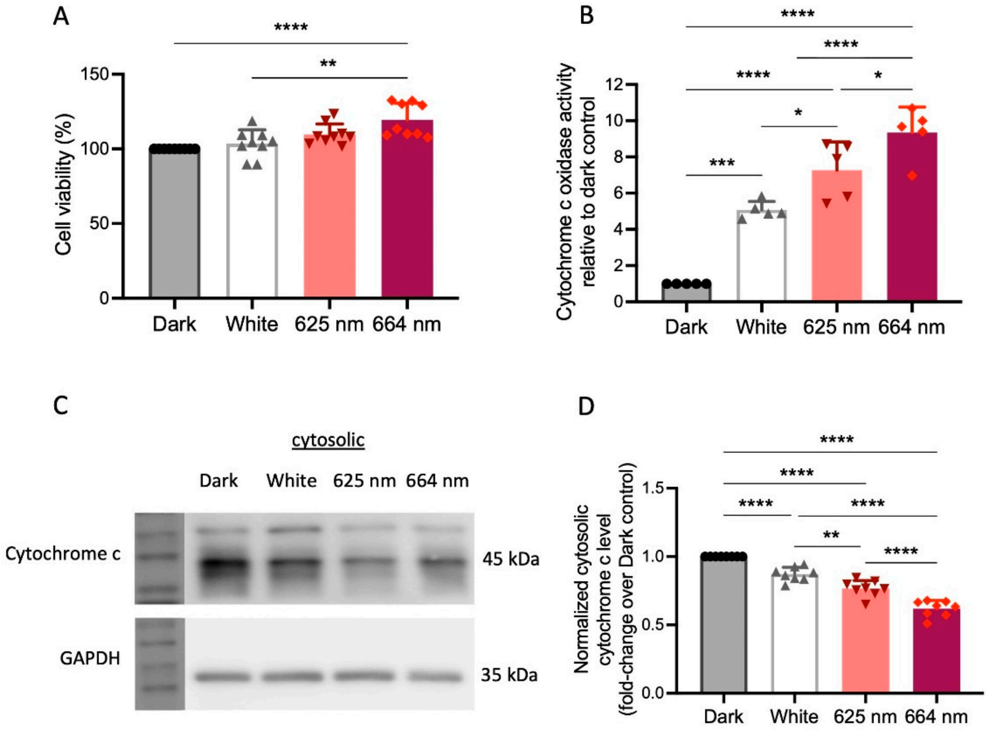

Figure S1. The assessment of (A) cell viability (n = 9), (B) CCO activity (n = 5), (C) a representative of eight individual western blots of cytochrome c expression in cytosolic fractions, and (D) the normalized cytochrome c protein level (n = 8) as shown in (C) in ARPE-19 cells. ARPE-19 cells were homogenized using glass beads (≤ 100 μm, 0.5 g per g cells; Sigma-Aldrich, Merck, Burlington, MA, USA) with 10 rounds of vigorous cell-breaking process, in which the cell mixture was vortexed for 1 min and subsequently chilled for 30 sec, and the cytosolic fractions from cells were isolated using subcellular fractionation. Data represent mean ± SD. The statistical significance was assessed by one-way ANOVA with Bonferroni post hoc test (**p* < 0.05, ***p* < 0.01, ****p* < 0.001, *****p* < 0.0001).

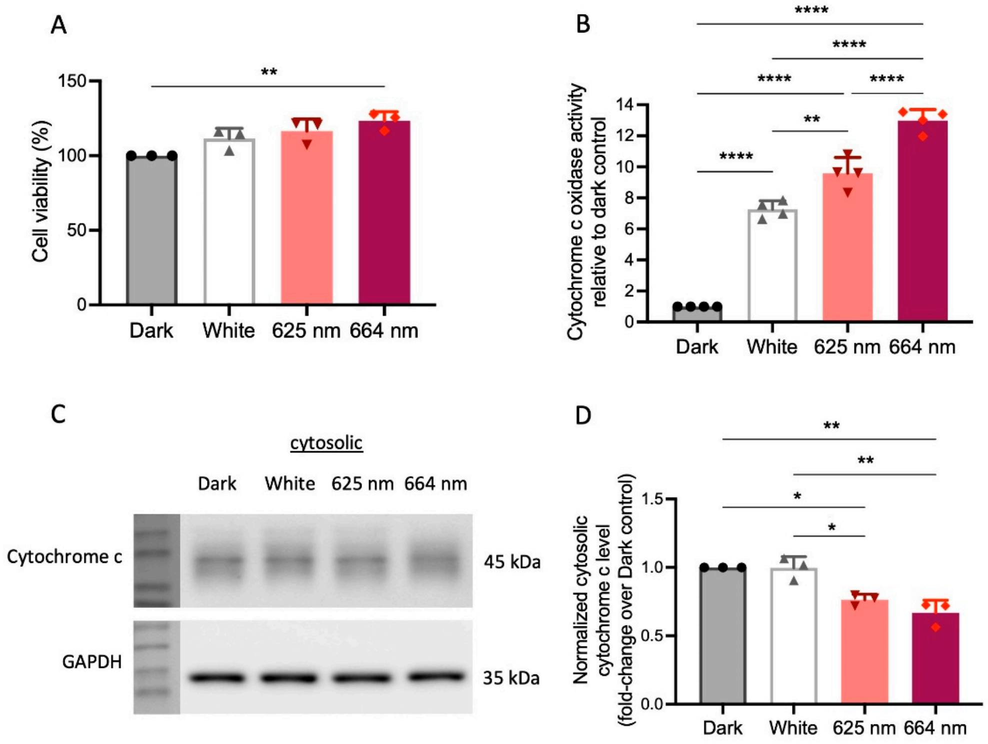

Figure S2. The assessment of (A) cell viability (n = 3), (B) CCO activity (n = 4), (C) a representative of three individual western blots of cytochrome c expression in cytosolic fractions extracted from RF/6A cells after photoirradiation, and (D) the normalized cytochrome c protein level (n = 3) as shown in (C) in RF/6A cells. RF/6A cells were homogenized using glass beads (≤ 100 µm, 0.5 g per g cells; Sigma-Aldrich, Merck, Burlington, MA, USA) with 10 rounds of a vigorous cell-breaking process, in which the cell mixture was vortexed for 1 min and subsequently chilled for 30 sec. The cytosolic fractions from the cells were isolated using subcellular fractionation. Data represent mean ± SD. The statistical significance was assessed by one-way ANOVA with Bonferroni post hoc test (*$p < 0.05$, **$p < 0.01$, ***$p < 0.001$, ****$p < 0.0001$).
